# The hepatocyte growth factor receptor (*MET)* gene is not associated with refractive error and ocular biometrics in a Caucasian population

**Published:** 2009-12-04

**Authors:** M. Schache, C.Y. Chen, M. Dirani, P.N. Baird

**Affiliations:** Centre for Eye Research Australia, University of Melbourne, Royal Victorian Eye and Ear Hospital, Melbourne, Australia.

## Abstract

**Purpose:**

The purpose of this study was to determine if genetic variants in the hepatocyte growth factor receptor (*MET)* gene are associated with refractive error and ocular biometric measures in a Caucasian cohort.

**Methods:**

A case-control association study using 818 Caucasian adults (37.2% male, 62.8% female; average age: 51.21±17.17 years) was undertaken. All individuals were genotyped for 16 tag single nucleotide polymorphisms (tSNPs) across the *MET* gene region. Myopia was defined as –0.5 DS or worse in both eyes and divided into high myopia (≤–6.0 DS) and low/moderate myopia (–0.5 DS to –5.99 DS). Hypermetropia was defined as at least +1.0 DS in both eyes. Genotyping results were analyzed using PLINK, comparing cases (all myopia, high myopia, low/moderate myopia, and hypermetropia) to controls (emmetropia). Association tests were also performed using the quantitative traits of refraction, axial length, anterior chamber depth, and corneal curvature.

**Results:**

No statistically significant genetic associations were detected for any of the 16 tSNPs with refractive error (myopia and hypermetropia) or ocular biometric measures.

**Conclusions:**

These data indicate there is likely no genetic association of the *MET* gene with myopia, axial length, anterior chamber depth, and corneal curvature in this cohort.

## Introduction

Myopia is a complex trait influenced by both genetic and environmental factors. It is a common condition affecting 16% to 22% of the adult population in Westernized regions such as Australia, the United States of America, and Europe [1]. The prevalence of myopia varies with race and age with rates reaching over 80% in urbanized regions of Singapore, China, and Japan [2,3]. The etiology of myopia is not fully understood but environmental factors such as excessive exposure to near work, high educational attainment and urbanization are contributing factors [4-6]. More recently, exposure to outdoor activity has been shown to be protective [6,7]. These known environmental factors account for 11% of the total phenotypic variance seen in myopia, suggesting that other factors are also involved [8]. There is also strong evidence from familial correlation and heritability studies that genetic factors are likely involved [9-13]. However, despite many loci having been identified, the exact nature of these genes remains unclear.

The hepatocyte growth factor (*HGF*) gene represents a strong biological candidate for myopia. It was initially described as a locus linked to eye weight in mice [14]. A genetic association has been reported in a Chinese cohort with high myopia as well as in two Caucasian cohorts with mild-moderate myopia [15-17]. This is the first myopia gene to be implicated in both high myopia and common myopia and the first to be definitively replicated in independent studies. Hence, to date, *HGF* represents one of the strongest candidate genes for myopia.

The biological functions of HGF are triggered upon binding to its receptor MET. HGF is the only known ligand for MET, and its binding results in the autophosphorylation of the receptor, which, in turn, activates a number of signaling cascades including the MAPK, PI3K STAT, and Wnt pathways [18]. The strong biological role of *HGF* in the development of myopia makes it not unreasonable to hypothesize that other genes in the HGF signaling cascade, such as *MET*, may also play a critical role in myopia. Recent genetic association studies into *MET* have reported mixed results showing a positive association with myopia reported in a Chinese cohort, but no association in a Caucasian population [16,19].

Although previous myopia genetic studies into *MET* have considered myopia as a binary trait, it is important to consider that myopia forms part of a continuous spectrum of refraction measurements. In addition, the refractive power of the eye is affected by several factors such as eye (axial) length and corneal structure; therefore, the definition of the phenotype must include axial length, anterior chamber depth, and corneal curvature measurements. The current study aims to address these issues by undertaking a case-control association study into *MET* using refraction and ocular biometric traits as submeasures of myopia.

## Methods

### Subjects

Individuals were selected from both the family and twin arms of the Genes in Myopia (GEM) Study [20,21]. The GEM Study consists of 785 individuals in 280 families in addition to 612 twin pairs. Individuals with a history of eye diseases, such as keratoconus or age-related macular degeneration, or a history of genetic disorders known to predispose to myopia, such as Stickler or Marfan syndromes, were excluded. Individuals with anisometropia of greater than a 2D difference between eyes were excluded, as were those of non-Caucasian ancestry and those with incomplete refraction measurements. For the families in the GEM Study, only index cases (probands) and unrelated married-ins were chosen unless they fell into the above categories, in which case an alternative family member, if available, was chosen. For the twins in the GEM Study, only one of each twin pair was chosen. The decision of which twin from each pair to choose was based on refraction status. In instances where one or both twins in a pair were myopic, the most myopic twin was chosen, and the co-twin was excluded. In instances where both twins in a pair were not myopic, one twin was chosen at random, and the co-twin was excluded. The choice of twin from each pair also took into account the selection criteria described above. The final cohort included 818 genetically unrelated individuals.

Refraction measurements for all individuals were obtained as described previously [20,21]. For the current study, the following phenotype definitions were used. Any myopia, low/moderate myopia, and high myopia were defined as ≤–0.5 DS, –0.499 DS to –5.99 DS, and ≤–6 DS, respectively. Hypermetropic individuals were defined by refraction measures ≥+1.0 DS and emmetropia as –0.5 DS to +1.0 DS. In total, this cohort consisted of 407 myopes (91 with high myopia and 316 with low/moderate myopia), 137 hypermetropes, and 274 emmetropes (controls).

DNA from all consenting individuals was collected from venous blood samples [22]. Written informed consent was obtained from all individuals prior to any testing, and ethics approval was provided by the Human Research and Ethics Committee of the Royal Victorian Eye and Ear Hospital (RVEEH), Melbourne. This study was conducted in accordance with the tenets of the Declaration of Helsinki.

### Tag Single Nucleotide Polymorphism (tSNP) Selection

Known SNPs encompassing the coding region of *MET* as well as the regions 2 kb upstream of the start codon and 2 kb downstream of the stop codon were identified from Phase III HapMap data (release 21). The CEU (also known as CEPH or Utah residents with ancestors from northern and western Europe) HapMap population was selected as the most representative of the current study cohort. The *Tagger* software was used to identify tSNPs in this gene utilizing a pairwise tagging approach, with the criteria of r^2^ > 0.8 and a minor allele frequency (MAF) >10%.

### Genotyping

All tSNPs were genotyped by the Australian Genome Research facility (Brisbane, Australia) using the MassArray platform and MALDI-TOF analysis (Sequenom, San Diego, CA).

### Statistical analysis

Genotyping data were assessed for deviations from Hardy-Weinberg equilibrium using PLINK [23]. Any SNPs that did not pass this test in controls (p < 0.05) were noted and excluded from further analysis. Association tests for myopia (all, high, and low/moderate) and hypermetropia as binary traits were performed using PLINK with the emmetropia group classed as controls. Association tests for the quantitative traits of refraction, axial length, corneal curvature, and anterior chamber depth were also performed using PLINK. All association tests included adjustments for multiple testing using the step-down Bonferroni correction. Haplotype analysis was also performed in PLINK using a two-, three-, four-, and five-marker sliding window. PLINK incorporates the standard expectation-maximization (EM) algorithm to statistically phase genotyping data. This algorithm estimates population-based haplotype probabilities based on maximum likelihood. The output from PLINK indicated all likely haplotypes, and any with a p < 0.05 were considered to be statistically significant. Power calculations were performed using Quanto version 1.2.4.

## Results

### Cohort demographics

A total of 818 individuals were recruited into this study, including 91 with high myopia (≤–6 DS), 316 with low/moderate myopia (–0.499 DS to –5.99 DS), 137 with hypermetropia (≥+1.0 DS), and 274 with emmetropia (–0.5 DS to +1.0 DS). The gender breakdown, mean ages, and mean refraction measures for these four groups are indicated in Table 1.

**Table 1:** Summary of demographics for the study population.

	Total cohort	All myopia (<-0.5 DS)	High myopia (≤ -6.00 DS)	Low/moderate myopia (-0.5 to -5.99 DS)	Hypermetropia (>+1.00 DS)	Emmetropia (<-0.05 to +1.00 DS)
N (total)	818	407	91	316	137	274
N (female: n (%))	514 (62.8)	242 (59.5)	48 (52.7)	194 (61.4)	97 (70.8)	175 (63.9)
Age (years: mean±stdev)	51.21±17.17	52.21±14.50	53.20±14.01	50.56±13.71	59.95±11.66	49.91±15.52
Refraction (DS: mean±stdev)	-1.64±3.09	-4.06±1.76	-7.75±1.76	-2.99±1.51	+1.96±0.97	+0.76±1.07

Refraction measures were available for the entire cohort with an average measurement of –1.64±3.09 DS (range: –15.13, 5.75) recorded. Axial length, anterior chamber depth, and corneal curvature measures were available for 718 individuals with average measurements of 24.13±1.47 mm (range: 17.24 mm to 30.79 mm), 3.49±0.40 mm (range: 1.95 mm to 5.63 mm), and 43.14±2.39 D (range: 34.31 D to 54.74 D), respectively. Measurements for all traits were highly correlated between the right and left eyes with correlation coefficients of 0.97, 0.97, 0.86, and 0.95 for refraction, axial length, anterior chamber depth, and corneal curvature, respectively. Hence, only measurements for the right eye were used in the analysis.

### SNP selection and genotyping

**Figure 1:**
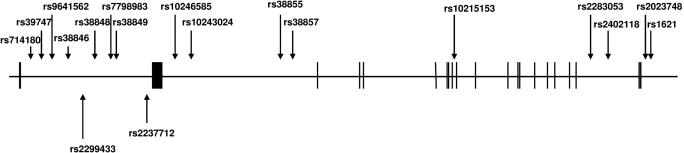
Exon-intron structure of the *MET* gene indicating the location of all tSNPs (above the line) and previously published myopia SNPs (below the line). Exons are indicated by boxes and are separated by introns indicated by solid lines.

**Figure 2:**
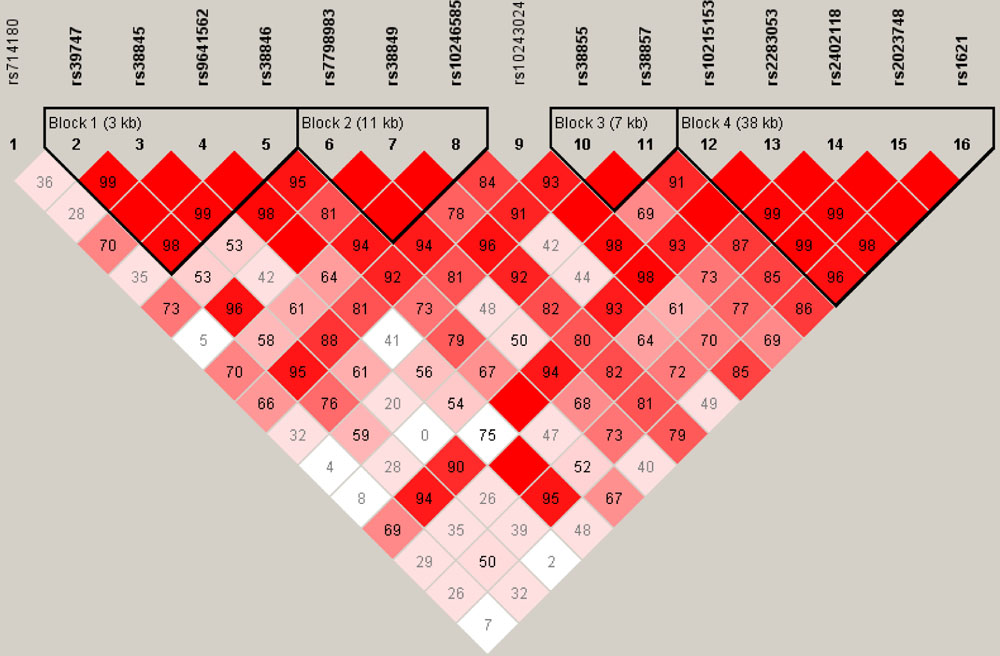
Linkage disequilibrium (LD) map for the 16 tSNPs genotyped for *MET* in the current study. Each diamond represents the correlation (r2) between each pair of SNPs with darker shades representing stronger linkage disequilibrium. The linkage disequilibrium block structure for the HapMap CEPH trios is indicated.

A total of 16 tSNPs in *MET* were chosen with a MAF >10%. All 16 tSNPs were located in the introns of *MET* with the physical locations indicated in Figure 1. The linkage disequilibrium block structure for *MET* from the HapMap CEU population is indicated in Figure 2. All 16 tSNPs were genotyped with an average SNP call rate of 96%. Of the 16 tSNPs, two (rs2402118 and rs2023748) were not in Hardy-Weinberg equilibrium in the emmetropic group (controls) and were therefore excluded from further analysis.

### Power calculations

A power calculation indicated that a cohort of this size has 80% power to detect an association with an odds ratio of 1.6, assuming a minor allele frequency of 10%, in the all myopia, high myopia, low/moderate myopia, and hypermetropia groups.

### Genetic associations tests

For the association tests with the binary traits of all myopia, high myopia, low/moderate myopia, and hypermetropia, a suggestive genetic association was found between high myopia and tSNP rs1621 (p=0.0126) and tSNP rs38857 (p=0.031), as can be seen in Table 2. Haplotype analysis using a two-, three-, four-, and five-marker sliding window did not indicate findings reaching statistical significance (data not shown). No genetic association with any of the tSNPs was detected for all myopia, low/moderate myopia, and hypermetropia. Association tests for the quantitative traits of refraction, axial length, anterior chamber depth, and corneal curvature showed a suggestive genetic association with refraction for tSNP rs38855 (p=0.049) as well as with corneal curvature with tSNP rs38846 (p=0.040; Table 3). No genetic associations were detected between axial length and anterior chamber depth and any of the tSNPs. The suggestive associations for high myopia, refraction, and corneal curvature did not remain statistically significant after multiple testing corrections.

**Table 2:** Association study results for all myopia, high myopia, low/moderate myopia and hypermetropia considered as binary traits.

SNP Name	Nucleotide position	Allele	All Myopia			High Myopia			Low/Moderate myopia			Hypermetropia		
			N	p*	OR (95% CI)	N	p*	OR (95% CI)	N	p*	OR (95% CI)	N	p*	OR (95% CI)
rs10215153	116186367	G	570	0.16	0.84 (0.66-1.07)	133	0.08	0.71 (0.48-1.04)	437	0.32	0.88 (0.68-1.13)	180	0.97	1.00 (0.73-1.37)
		A	228			45			183			86		
rs10243024	116133839	G	528	0.90	0.98 (0.74-1.30)	114	0.77	1.07 (0.69-1.63)	414	0.78	0.95 (0.71-1.28)	174	0.21	1.25 (0.87-1.77)
		A	162			38			124			68		
rs10246585	116131334	T	578	0.98	1.00 (0.78-1.28)	123	0.46	1.15 (0.79-1.67)	455	0.74	0.95 (0.73-1.24)	179	0.15	1.26 (0.91-1.74)
		C	216			53			163			85		
rs1621	116224842	A	563	0.11	0.83 (0.65-1.04)	136	0.01	0.61 (0.41-0.90)	427	0.37	0.89 (0.69-1.14)	175	0.75	1.05 (0.77-1.43)
		G	235			42			193			93		
rs2283053	116214255	A	635	0.98	1.00 (0.76-1.32)	132	0.14	1.35 (0.90-2.02)	503	0.54	0.91 (0.67-1.22)	206	0.36	1.18 (0.82-1.68)
		G	157			44			113			60		
rs38845	116109038	G	418	0.33	0.90 (0.72-1.12)	81	0.38	1.16 (0.82-1.63)	337	0.12	0.83 (0.66-1.05)	127	0.72	1.05 (0.78-1.41)
		A	378			95			283			135		
rs38846	116111767	T	652	0.28	0.86 (0.65-1.13)	144	0.59	0.89 (0.57-1.37)	508	0.28	0.84 (0.63-1.14)	214	0.87	0.96 (0.67-1.40)
		C	140			32			108			52		
rs38849	116119775	G	605	0.62	0.94 (0.73-1.21)	144	0.26	0.79 (0.52-1.19)	461	0.90	0.98 (0.75-1.28)	200	0.82	0.96 (0.68-1.35)
		C	189			38			151			64		
rs38855	116145280	G	401	0.13	1.18 (0.95-1.47)	91	0.24	1.23 (0.87-1.72)	310	0.18	1.17 (0.92-1.47)	110	0.18	0.81 (0.60-1.09)
		A	397			87			310			158		
rs38857	116152649	C	595	0.16	0.84 (0.65-1.07)	141	0.03	0.64 (0.42-0.96)	454	0.43	0.90 (0.69-1.16)	188	0.83	1.03 (0.75-1.42)
		T	205			37			168			80		
rs39747	116108275	T	431	0.30	1.12 (0.90-1.41)	102	0.72	0.94 (0.66-1.33)	329	0.16	1.18 (0.93-1.49)	154	0.61	0.92 (0.68-1.25)
		C	355			70			285			104		
rs714180	116106228	G	426	0.92	0.99 (0.79-1.23)	89	0.49	1.12 (0.80-1.58)	337	0.68	0.95 (0.75-1.20)	154	0.23	0.83 (0.62-1.12)
		A	374			89			285			114		
rs7798983	116119433	A	534	0.60	0.94 (0.73-1.20)	111	0.77	1.06 (0.72-1.54)	423	0.45	0.90 (0.69-1.17)	169	0.27	1.19 (0.86-1.63)
		G	226			53			173			91		
rs9641562	116110027	A	753	0.31	0.80 (0.51-1.23)	172	0.06	0.45 (0.18-1.06)	581	0.65	0.89 (0.57-1.41)	252	0.49	0.80 (0.44-1.47)
		C	47			6			41			16		

* p-values that have not been corrected for Type 1 errors are shown. Application of the step-down Bonferroni correction method means that a p-value cut-off of 0.004 (0.05/14) represents statistical significance for this study.

**Table 3:** Association test results for refraction, axial length (AL), anterior chamber depth (ACD) and corneal curvature (CC) when considered as quantitative traits.

SNP name	Nucleatide position	Refraction (p*)	Axial length (p*)	ACD (p*)	CC (p*)
rs10215153	116186367	0.26	0.13	0.76	0.35
rs10243024	116133839	0.26	0.05	0.64	0.94
rs10246585	116131334	0.69	0.05	0.78	0.22
rs1621	116224842	0.06	0.15	0.65	0.29
rs2283053	116214255	0.91	0.09	0.79	0.92
rs38845	116109038	0.86	0.08	0.70	0.52
rs38846	116111767	0.94	0.04	0.34	0.04
rs38849	116119775	0.62	0.03	0.34	0.40
rs38855	116145280	0.05	0.08	0.22	0.68
rs38857	116152649	0.15	0.10	0.56	0.91
rs39747	116108275	0.36	0.05	0.60	0.70
rs714180	116106228	0.17	0.04	0.53	0.94
rs7798983	116119433	0.54	0.06	0.84	0.85
rs9641562	116110027	0.63	0.23	0.41	0.76

* p-values that have not been corrected for Type 1 errors are shown. Application of the step-down Bonferroni correction method means that a p-value cut-off of 0.004 (0.05/14) represents statistical significance for this study.

Of the 407 myopic individuals, the age of onset of myopia was known for 343 (84.2%) individuals, of whom 241 had childhood onset myopia and 102 had adult onset myopia. Association tests for all myopia, high myopia, low/moderate myopia, and hypermetropia were repeated in each of these groups, but no significant p-values were detected (data not shown).

## Discussion

This study examined the genetic association of the *MET* gene with refractive error (low/moderate myopia, high myopia, and hypermetropia) as well as the quantitative measures of refraction, axial length, corneal curvature, and anterior chamber depth. Using a tag SNP approach, we captured all the common genetic variants within the coding and promoter regions of *MET* by genotyping 16 tSNPs. We found no association with low/moderate myopia, high myopia, and hypermetropia in a Caucasian cohort. Additional exploration of the quantitative ocular biometric measures showed no genetic association in this cohort. By performing a detailed analysis of myopia and its underlying subphenotypes, we were able to exclude *MET* as a myopia gene in this cohort.

Recent genetic association studies into *MET* have reported conflicting results. Analysis of a Singaporean cohort suggested that the intronic SNP rs2073560 was associated with moderate myopia and with an increased rate of change of myopia in children aged 7-12 years [19]. However, no genetic association was found with axial length and hypermetropia. By contrast, analysis of a Caucasian adult cohort indicated that there was no genetic association between high or low/moderate myopia and *MET* [15]. The current study did not directly genotype rs20702560, but instead used the proxy tag SNP rs2283053, which is in complete linkage disequilibrium with rs2073560. The current study was also performed in a Caucasian cohort, but differed from the Yankovitch et al. [15] study in that hyperopia was also considered and refraction was also analyzed as a quantitative trait. Moreover, axial length, corneal curvature, and anterior chamber depth were also analyzed, none of which were reported in the Yankovitch et al. [15] study.

Although our findings have not indicated that *MET* plays a direct genetic role in myopia and hypermetropia, we cannot rule out the possibility that additional genes in related molecular pathways may also be involved. This is supported by the fact that we and others have found the *HGF* gene, which codes for the primary ligand for *MET*, to be associated with both high and low/moderate myopia [15,16]. However, given that MET is the only receptor for HGF, the relationship between HGF and these genes is likely complex.

The study design used for the current work has many strengths, including the choice of cohorts and the tag SNP approach. The cohort was chosen to include a homogenous population of Caucasian ancestry, which allowed minimization of potential effects of population admixture. The tag SNP approach is also a strength because it allows maximal coverage of the *MET* gene and its promoter region. However, since the tag SNPs were chosen to include common SNPs (MAF > 0.1), it is possible that this methodology excluded rare variants that may also be implicated in refractive error. To determine if this is the case, additional sequencing of the entire coding region of *MET* would be necessary.

Through a detailed and thorough analysis of refractive error and the underlying subphenotypes, the current study has been able to make a strong case suggesting it is unlikely that *MET* plays a direct genetic role in refractive error. This suggests that the relationship between known candidate genes, such as *HGF*, and other candidate genes is indeed complex, and much more work is required to unravel the nature of these relationships.
